# Extreme umbilical cord lengths, cord knot and entanglement: Risk factors and risk of adverse outcomes, a population-based study

**DOI:** 10.1371/journal.pone.0194814

**Published:** 2018-03-27

**Authors:** Lorentz Erland Linde, Svein Rasmussen, Jörg Kessler, Cathrine Ebbing

**Affiliations:** 1 Department of Obstetrics and Gynecology, Haukeland University Hospital, Bergen, Norway; 2 Department of Clinical Science, University of Bergen, Bergen, Norway; Helsingin Yliopisto, FINLAND

## Abstract

**Objectives:**

To determine risk factors for short and long umbilical cord, entanglement and knot. Explore their associated risks of adverse maternal and perinatal outcome, including risk of recurrence in a subsequent pregnancy. To provide population based gestational age and sex and parity specific reference ranges for cord length.

**Design:**

Population based registry study.

**Setting:**

Medical Birth Registry of Norway 1999–2013.

**Population:**

All singleton births (gestational age>22weeks<45 weeks) (n = 856 300).

**Methods:**

Descriptive statistics and odds ratios of risk factors for extreme cord length and adverse outcomes based on logistic regression adjusted for confounders.

**Main outcome measures:**

Short or long cord (<10^th^ or >90^th^ percentile), cord knot and entanglement, adverse pregnancy outcomes including perinatal and intrauterine death.

**Results:**

Increasing parity, maternal height and body mass index, and diabetes were associated with increased risk of a long cord. Large placental and birth weight, and fetal male sex were factors for a long cord, which again was associated with a doubled risk of intrauterine and perinatal death, and increased risk of adverse neonatal outcome. Anomalous cord insertion, female sex, and a small placenta were associated with a short cord, which was associated with increased risk of fetal malformations, placental complications, caesarean delivery, non-cephalic presentation, perinatal and intrauterine death. At term, cord knot was associated with a quadrupled risk of perinatal death. The combination of a cord knot and entanglement had a more than additive effect to the association to perinatal death. There was a more than doubled risk of recurrence of a long or short cord, knot and entanglement in a subsequent pregnancy of the same woman.

**Conclusion:**

Cord length is influenced both by maternal and fetal factors, and there is increased risk of recurrence. Extreme cord length, entanglement and cord knot are associated with increased risk of adverse outcomes including perinatal death. We provide population based reference ranges for umbilical cord length.

## Introduction

A normal umbilical cord is of obvious importance for a normal fetal development. It has been estimated that about 10% of intrauterine deaths in the USA may be attributable to umbilical cord complications, and these complications are associated with clinically significant placental pathology [1]. Lately there has been an increased awareness of placental and cord abnormalities and their associated risk of adverse outcome for the mother and the newborn [2–4]. In case studies excessive long cords have been associated with cord entanglements, emergency deliveries and fetal thrombotic vasculopathy in the placenta, fetal death and increased risk of neurological complications [5,6]. A short cord has been associated with increased risk of fetal malformations, fetal distress and possibly placental abruption [7–9]. Although anomalous cord length is associated with adverse outcome [5, 9], normal cord length is poorly defined in many studies, and population based studies and reference ranges are lacking. Also studies on risk factors and outcome of cord entanglement and knots are scarce, and population studies are yet to be performed. Therefore, the aims of the present study was 1: to determine risk factors for long and short umbilical cord, cord knots and entanglements, 2: to study the associated risks of adverse outcome of pregnancies with abnormal cord length, cord knot and entanglement in the Norwegian population, 3: to study the risk of recurrence of abnormal cord length, cord knot and entanglement in a subsequent pregnancy of the same woman, and 4: to provide population based gestational age, sex and parity specific charts for umbilical cord length.

## Methods

We performed a population-based register study of all singleton births in Norway with gestational age >21 weeks and <45 weeks during the period 1999–2013 (*n* = 856 300) using data from the Medical Birth Register of Norway (MBRN). The attending midwife or physician performed the examinations of the neonate, placenta, membranes and cord, and entered the requested information into a registration form shortly after delivery. Information regarding the umbilical cord has been specified since 1999 using tick boxes named: “normal, marginal, velamentous, vessel anomalies, entanglement (around the neck or other body parts) and cord knot”. The length of the umbilical cord was measured in centimetres. Placenta with cord and membranes attached were weighed in grams. To construct empirical percentiles of cord length for the population we included cord lengths from 1 to 290 cm (*n* = 797 096). The attending midwife or physician also clinically estimated the amount of amniotic fluid (poly- or oligohydramnios) and postpartum bleeding volume. Preterm pre labour rupture of the membranes (PPROM) was defined as rupture of the membranes <37 weeks of gestation and >24 hours before birth (yes/no). Gestational age was based on ultrasound dating in the first half of pregnancy when available (in 97.0% of the cases) or the mother’s last menstrual period. Preterm birth was defined as birth before gestational week 37. Parity was defined as the number of previous deliveries. From 2006 maternal weight and height from the pregnancy file has been included in the register. Body mass index was available for 37.2% of the pregnancies from 2006 (*n* = 174 337 of 468 321 possible).

Pregnancies conceived by assisted reproductive technology (ART) have been notified in the register on voluntary basis from 1988 and compulsory basis since 2001 (*n* = 16 810). The diagnosis of abruption of the placenta and placenta previa was done by the clinician.

All neonates were examined by a physician who recorded any malformation at birth or at the neonatal care unit. Severe malformations were defined by specific Q diagnoses in the International Classification of Diseases (10^th^ revision) system (see supporting information). Transferral to neonatal intensive care unit was registered.

Long (>90^th^ gestational age, sex and parity (0 and 1+) specific empirical percentile) or short (<10^th^ empirical percentile) umbilical cord, umbilical cord knot or entanglement were considered as outcome measures, as well as exposures. Placenta previa, abruption of the placenta, preeclampsia, caesarean delivery, non-cephalic presentation, low Apgar score at 5 minutes, transferal to neonatal intensive care unit (NICU), malformations, birth weight- and placental weight (empirical gestational, sex and parity specific percentiles), intrauterine and perinatal death were considered as outcomes of these (cord) exposures.

Variables were included in the model according to their potential influence on the risk estimates: parity, maternal and paternal age, neonatal sex, maternal BMI on the first prenatal visit, maternal height, cigarette smoking at the beginning of pregnancy, maternal medical conditions, anomalous cord insertion site on the placenta, conception by ART, small or large birth weight and placental weight for gestational age based on empirical percentiles for the population (birthweight <10^th^ or >90^th^ percentile, SGA and LGA), and low or high placental weight(<10^th^ or >90^th^ percentile).

In order to study trend we categorized the study period in 5-year intervals (1999–2003, 2004–2008, and 2009–2013).

The Regional Committee for Medical and Health Research Ethics West (REK Vest) approved the study protocol (approval no. REC West 2011/949) and waived the need for written informed consent from the participants due to the data being analysed anonymously.

The data are reported in accordance with the STROBE guidelines (https://www.strobe-statement.org).

### Statistics

Odds ratios (ORs) and 95% confidence intervals (95%CI) for short and long umbilical cord, cord knot and cord entanglement were estimated using Generalized Estimating Equations analyses, with adjustments for possible confounding factors. We analysed the data with the population stratified for gestational age at birth in weeks: 22–27, 28–36, 37–41, 42+, or in preterm (gestational age below 37 weeks) and term (≥37 weeks) births. In order to calculate ORs for a repeat long or short cord length, cord knot and entanglement in the subsequent pregnancy, data from the first and second births of each woman were linked using national identification numbers. Differences within the population were assessed by chi-squared test, and p<0.05 was defined significant.

We calculated gestational age (weeks), parity (0, 1+) and sex specific empirical umbilical cord length percentiles, but in analyses where fetal sex and/ or parity was included in the model, gestational age specific percentiles (not specific for sex or parity) for cord length were used. Below 29 weeks linear regression within strata of whole gestational age weeks, with umbilical cord length as outcome and gender and parity (0, 1+) as independent variables, revealed non-significant influence of sex and parity. Thus, the percentile tables were made sex and parity specific for gestational age above 28 weeks only. The percentiles were smoothed by Kernel smoothing (SigmaPlot version 13.0 (Systat Software, San Jose, CA)). Statistical Package for the Social Sciences for Windows (version 24; SPSS, Chicago IL, USA) was used for the statistical analyses.

### Results

Descriptive information of the study population according to properties of the cord (long or short cord, cord knot and entanglement) is shown in Table 1.

**Table 1:** Maternal and pregnancy characteristics for pregnancies with long or short (>90th percentile or <10th percentile) umbilical cord, cord knot and entanglement in the population of singleton births in Norway 1999–2013.

			Long cord (>90th percentile)			Short cord (<10th percentile)			Cord knot				Cord entanglement		
Characteristics		total	n	%	p-value	n	%	p-value	total	n	%	p-value	n	%	p-value
**Maternal age (years)**	<20	17649	1341	7.60	<0.001	1637	9.28	<0.001	19168	181	0.94	<0.001	4164	21.72	<0.001
	20–24	117980	9315	7.90		10977	9.30		126936	1238	0.98		27225	21.45	
	25–29	259300	21138	8.15		23517	9.07		278348	3158	1.13		58546	21.03	
	30–34	263366	23375	8.88		22285	8.46		282562	4050	1.43		57663	20.41	
	35–39	117707	11644	9.89		9107	7.74		126499	2180	1.72		25033	19.79	
	40 +	21029	2246	10.68		1679	7.98		22714	463	2.04		4320	19.02	
	Total	797031	69059	8.66		69202	8.68		856227	11270	1.32		176951	20.67	
**Parity**	0	331108	23861	7.21	<0.001	35451	10.71	<0.001	354902	3038	0.86	<0.001	70258	19.80	<0.001
	1	285462	24740	8.67		22598	7.92		306427	4210	1.37		64334	20.99	
	2	127640	14018	10.98		8115	6.36		137557	2680	1.95		30119	21.90	
	3	35832	4379	12.22		2063	5.76		38875	888	2.28		8303	21.36	
	4+	17054	2064	12.10		984	5.77		18539	455	2.45		3943	21.27	
	Total	797096	69062	8.66		69211	8.68		856300	11271	1.32		176957	20.67	
**Fetal gender**	Boy	409410	40353	9.86	<0.001	30470	7.44	<0.001	439407	6907	1.57	<0.001	95408	21.71	<0.001
	Girl	387654	28705	7.40		38732	9.99		416631	4364	1.05		81543	19.57	
**ART**	No	781178	69162	8.85	NS	67998	8.70	<0.001	839490	11097	1.32	0.0012	173798	20.70	<0.001
	Yes	15886	1437	9.05		1563	9.84		16810	174	1.04		3159	18.79	
**BMI at the beginning of pregnancy**	<18.5	6949	407	5.86	<0.001	765	11.01	<0.001	7215	63	0.87	<0.001	1567	21.72	NS
	18.5–24.9	102781	7966	7.75		9787	9.52		106680	1145	1.07		23810	22.32	
	25–29.9	37733	3880	10.28		2935	7.78		39196	520	1.33		8900	22.71	
	>30	20430	2619	12.82		1364	6.68		21246	328	1.54		4844	22.80	
**Total**		167893	14872	8.86		14851	8.85		174337	2056	1.18		39121	22.44	
**Smoking**	No	562309	50364	8.96	<0.001	48772	8.67	<0.001	599743	7923	1.32	<0.001	129881	21.66	<0.001
	Sometimes	12949	1235	9.54		1034	7.99		14004	181	1.29		2990	21.35	
	Daily	94397	8613	9.12		7764	8.22		103122	1578	1.53		24332	23.60	
	Not answered	127409	10387	8.15		11991	9.41		139431	1589	1.14		19754	14.17	
**Maternal height (cm)**	<150	818	41	5.01	<0.001	55	6.72	<0.001	849	4	0.47	<0.001	159	18.73	<0.001
	150–159	20432	1441	7.05		1140	5.58		21287	199	0.93		4598	21.60	
	160–169	96836	8050	8.31		4204	4.34		100653	1146	1.14		22771	22.62	
	170–179	60492	6044	9.99		2122	3.51		62756	825	1.31		14213	22.65	
	180+	4492	562	12.51		141	3.14		4642	65	1.40		1061	22.86	
**Pre gestational diabetes mellitus**	No	791627	69807	8.82	<0.001	69287	8.75	<0.001	850402	11165	1.31	0.0011	176046	20.70	<0.001
	yes	5437	792	14.57		274	5.04		5898	106	1.80		911	15.45	
**Gestational diabetes mellitus**	No	786161	69169	8.80	<0.001	68926	8.77	<0.001	844856	11063	1.31	<0.001	174636	20.67	NS
	Yes	10903	1430	13.12		635	5.82		11444	208	1.82		2321	20.28	
**Pre-existing hypertension**	No	792749	70142	8.85	<0.001	33030	4.17	NS	851518	11180	1.31	<0.001	175953	20.66	NS
	Yes	4315	457	10.59		167	3.87		4782	91	1.90		1004	21.00	
**Marginal cord insertion**	No	751043	66684	8.88	0.006	30873	4.11	<0.001	807183	10699	1.33	0.0024	164075	20.33	<0.001
	Yes	46021	3915	8.51		2324	5.05		49117	572	1.16		12882	26.23	
**Velamentous cord insertion**	No	785000	69546	8.86	NS	32393	4.13	<0.001	843315	11149	1.32	<0.001	173729	20.60	<0.001
	Yes	12064	1053	8.73		804	6.66		12985	122	0.94		3228	24.86	
**Placental weight<10th percentile**	No	718903	67385	9.37	<0.001	56142	7.81	<0.001	753533	10297	1.37	<0.001	157099	20.85	NS
	Yes	71597	2561	3.58		12661	17.68		75705	683	0.90		15629	20.64	
**Placental weight>90th percentile**	No	714228	56521	7.91	<0.001	65594	9.18	<0.001	749473	9457	1.26	<0.001	156705	20.91	<0.001
	Yes	76272	13425	17.60		3209	4.21		79765	1523	1.91		16023	20.09	
**Birth weight<10th percentile**	No	718181	65525	9.12	<0.001	57658	8.03	<0.001	771324	10113	1.31	NS	156844	20.33	<0.001
	Yes	78449	5039	6.42		11842	15.10		84164	1151	1.37		20005	23.77	
**Birth weight>90th percentile**	No	718427	59503	8.28	<0.001	65772	9.16	<0.001	771107	10026	1.30	<0.001	162763	21.11	<0.001
	Yes	78203	11061	14.14		3728	4.77		84381	1238	1.47		14086	16.69	
**Year of birth**	1999–2003	246159	22503	9.14	<0.001	21019	8.54	<0.001	277673	4146	1.49	<0.001	63255	22.78	<0.001
	2009–2013	265475	23231	8.75		22866	8.61		282466	3778	1.34		54541	19.31	
	2009–2013	285430	24865	8.71		25676	9.00		296161	3347	1.13		59161	19.98	
**Gestational age at birth (weeks)**	22–27	2538	216	8.51	0.002	200	7.88	0.007	3320	41	1.23	<0.001	232	6.99	<0.001
	28–36	39943	3663	9.17		3316	8.30		43459	652	1.50		6463	14.87	
	37–41	701315	61819	8.81		61416	8.76		751904	9900	1.32		157056	20.89	
	42+	53268	4901	9.20		4629	8.69		57617	678	1.18		13206	22.92	
**Oligohydramnios**	No	774296	68320	8.82	<0.001	67267	8.69	<0.001	832171	10922	1.31	NS	171056	20.56	<0.001
	Yes	22768	2279	10.01		2294	10.08		24129	349	1.45		5901	24.46	
**Polihydramnios**	No	788851	69714	8.84	<0.001	68880	8.73	NS	847568	11103	1.31	<0.001	175166	20.67	NS
	Yes	8213	885	10.78		681	8.29		8732	168	1.92		1791	20.51	
**Malformations**	No	761515	67761	8.90	<0.001	65435	8.59	<0.001	818253	10756	1.31	<0.001	170217	20.80	<0.001
	Yes	35549	2838	7.98		4126	11.61		38047	515	1.35		6740	17.71	

ART; assisted reproductive technology, BMI: Body mass index,

Including maternal age and parity in the models did not significantly influence the associations (Tables 2 and 3). Therefore only unadjusted ORs are given in the tables, and exceptions are specified in the text. In the tables we report only significant findings.

**Table 2:** Odds ratios of a long cord (>90^th^ percentile), short cord (<10^th^ percentile), cord knot and entanglement according to maternal and pregnancy characteristics in singletons in Norway 1999–2013.

Outcome Condition		Long cord (>90^th^ percentile)			Short cord(<10^th^ percentile)			Cord knot			Cord entanglement		
Exposure		OR	95%CI		OR	95%CI		OR	95%CI		OR	95%CI	
**Parity**	0	reference											
	1	1.22	1.20	1.24	0.72	0.80	0.73	1.61	1.54	1.69	1.07	1.06	1.09
	2	1.59	1.55	1.62	0.57	0.55	0.58	2.30	2.18	2.43	1.13	1.12	1.15
	3	1.79	1.73	1.86	0.51	0.49	0.53	2.71	2.51	2.92	1.09	1.07	1.13
	4+	1.77	1.69	1.86	0.51	0.48	0.55	2.91	2.64	3.22	1.09	1.06	1.13
**Maternal age (years)**	<20	reference											
	20–24	NS			NS			NS			NS		
	25–29	1.08	1.02	1.14	NS			1.20	1.04	1.40	0.96	0.93	0.99
	30–34	1.18	1.12	1.25	0.90	0.86	0.95	1.53	1.31	1.77	0.92	0.89	0.96
	35–39	1.34	1.26	1.42	0.82	0.78	0.87	1.84	1.58	2.14	0.89	0.86	0.92
	40 +	1.45	1.35	1.56	0.85	0.79	0.91	2.18	1.84	2.59	0.85	0.81	0.89
**Fetal gender**	Boy	reference											
	Girl	0.73	0.72	0.74	1.38	1.36	1.40	0.66	0.64	0.69	0.88	0.87	0.89
**ART**	Yes	NS			1.14	1.09	1.21	0.78	0.67	0.91	0.89	0.85	0.92
**BMI**	<18.5	reference											
	18.5–24.9	1.35	1.22	1.50	0.85	0.79	0.92	NS			NS		
	25–29.9	1.84	1.66	2.05	0.68	0.63	0.74	1.53	1.17	1.98	1.06	1.00	1.13
	>30	2.36	2.12	2.63	0.58	0.53	0.63	1.78	1.36	2.33	1.06	1.00	1.14
**Smoking at the beginning of pregnancy**	no	reference											
	sometimes	1.07	1.01	1.14	0.91	0.86	0.97	NS			NS		
	daily	1.02	1.00	1.05	0.94	0.92	0.97	1.16	1.10	1.23	1.12	1.11	1.15
	na	0.90	0.88	0.92	1.09	1.07	1.12	0.84	0.79	0.98	0.57	0.56	0.58
**Maternal height (cm)**	160–169	reference											
	<150	0.59	0.43	0.81	1.60	1.31	1.96	NS			0.79	0.66	0,94
	150–159	0.82	0.78	0.87	1.25	1.19	1.31	0.82	0.70	0.95	0.94	0.91	0.98
	170–179	1.24	1.20	1.28	0.87	0.82	0.79	1.16	1.06	1.27	NS		
	180+	1.51	1.38	1.66	0.72	0.64	0.81	NS			NS		
**Pre-gestational diabetes mellitus**	yes	1.76	1.63	1.90	0.55	0.49	0.62	1.38	1.13	1.67	0.70	0.65	0.75
**Gestational diabetes mellitus**	Yes	1.56	1.48	1.66	0.64	0.59	0.70	1.40	1.21	1.60	NS		
**Pre-existing hypertension**	Yes	1.22	1.11	1.35	0.86	0.77	0.97	1.46	1.18	1.80	NS		
**Marginal cord insertion**	Yes	0.95	0.92	0.99	1.20	1.16	1.23	0.88	0.81	0.95	1.39	1.37	1.42
**Velamentous cord insertion**	Yes	NS			1.49	1.41	1.57	0.71	0.59	0.85	1.28	1.22	1.33
**Placental weight<10th percentile**	Yes	0.36	0.34	0.37	2.54	2.48	2.59	0.66	0.61	0.71	NS		
**Placental weight>90th percentile**	Yes	2.49	2.44	2.54	0.43	0.42	0.45	1.52	1.44	1.61	0.95	0.93	0.97
**Birth weight<10th percentile**	Yes	0.68	0.66	0.70	2.04	1.99	2.08	NS			1.22	1.20	1.24
**Birth weight>90th percentile**	Yes	1.82	1.79	1.86	0.50	0.48	0.51	1.13	1.07	1.20	0.75	0.73	0.76
**Year of birth**	1999–2003	reference											
	2009–2013	0.95	0.93	0.97	NS			0.89	0.86	0.94	0.81	0.80	0.82
	2009–2013	0.95	0.93	0.97	1.06	1.04	1.08	0.75	0.72	0.79	0.85	0.84	0.86
**Gestational age at birth (weeks)**	22–27	reference											
	28–36	NS			NS			NS			2.33	2.03	2.66
	37–41	NS			NS			NS			3.51	3.07	4.02
	42+	NS			NS			NS			3.96	3.46	4.53
**Oligohydramnios**	Yes	1.15	1.10	1.20	1.18	1.13	1.23	NS			1.25	1.21	1.29
**Polyhydramnios**	Yes	1.25	1.16	1.34	NS			1.48	1.27	1.72	NS		

ART; assisted reproductive technology, BMI: Body mass index, OR; Odds ratio, CI; Confidence Interval, NS; non-significant

**Table 3:** Odds ratios of adverse pregnancy outcomes in pregnancies with a short umbilical cord (<10th sex and parity specific percentile) in the population of singleton births in Norway 1999–2013.

Exposure	Outcome						
Cord <10th percentile	Condition	Yes (n)	Total (n)	%	OR	95% CI	
No	Malformation	31456	727885	4.32			
Yes		4100	69211	5.92	1.40	1.35	1.44
No	Placenta previa	2023	727885	0.28			
Yes		275	69211	0.40	1.50	1.33	1.70
No	Abruptio placentae	2479	727885	0.34			
Yes		366	69211	0.53	1.54	1.38	1.72
No	Post partum haemorrhage	117369	727503	16.08			
Yes		11757	69211	16.99	1.03	1.00	1.05
No	Manual removal of the placenta	9859	727885	1.35			
Yes		1260	69211	1.82	1.30	1.23	1.38
No	Preeclampsia	25250	727885	3.47			
Yes		2253	69211	3.26	0.85	0.82	0.89
No	PPROM^¥^	6203	727885	0.85			
Yes		357	69211	0.52	0.65	0.58	0.72
No	Preterm birth	39223	727885	5.39			
Yes		3287	69211	4.75	0.85	0.82	0.89
No	Transverse lie	2100	727885	0.29			
Yes		227	69211	0.33	1.20	1.05	1.37
No	Breech position	24290	727885	3.34			
Yes		4091	69211	5.91	1.74	1.68	1.80
No	Emergency caesarean	63155	727885	8.68			
Yes		9961	69211	14.39	1.65	1.61	1.68
No	Caesarean delivery	104329	727885	14.33			
Yes		15787	69211	22.81	1.73	1.70	1.77
No	Perinatal death	3332	727885	0.46			
Yes		465	69211	0.67	1.50	1.36	1.65
No	Intrauterine death	2539	727885	0.35			
Yes		325	69211	0.47	1.37	1.22	1.53
No	NICU*	55866	727885	7.68			
Yes		5847	69211	8.45	1.10	1.07	1.13
No	5 minute Apgar score <7	19201	727885	2.64			
Yes		2186	69211	3.16	1.15	1.10	1.21

^¥^ Preterm pre labor rupture of the membranes

* Transferral to neonatal intensive care unit

OR; Odds ratio, CI; Confidence Interval

#### What influences the length of the cord?

There was an overall slight reduction in the risk of a long cord during the study period (Table 2).

**Fig 1:**
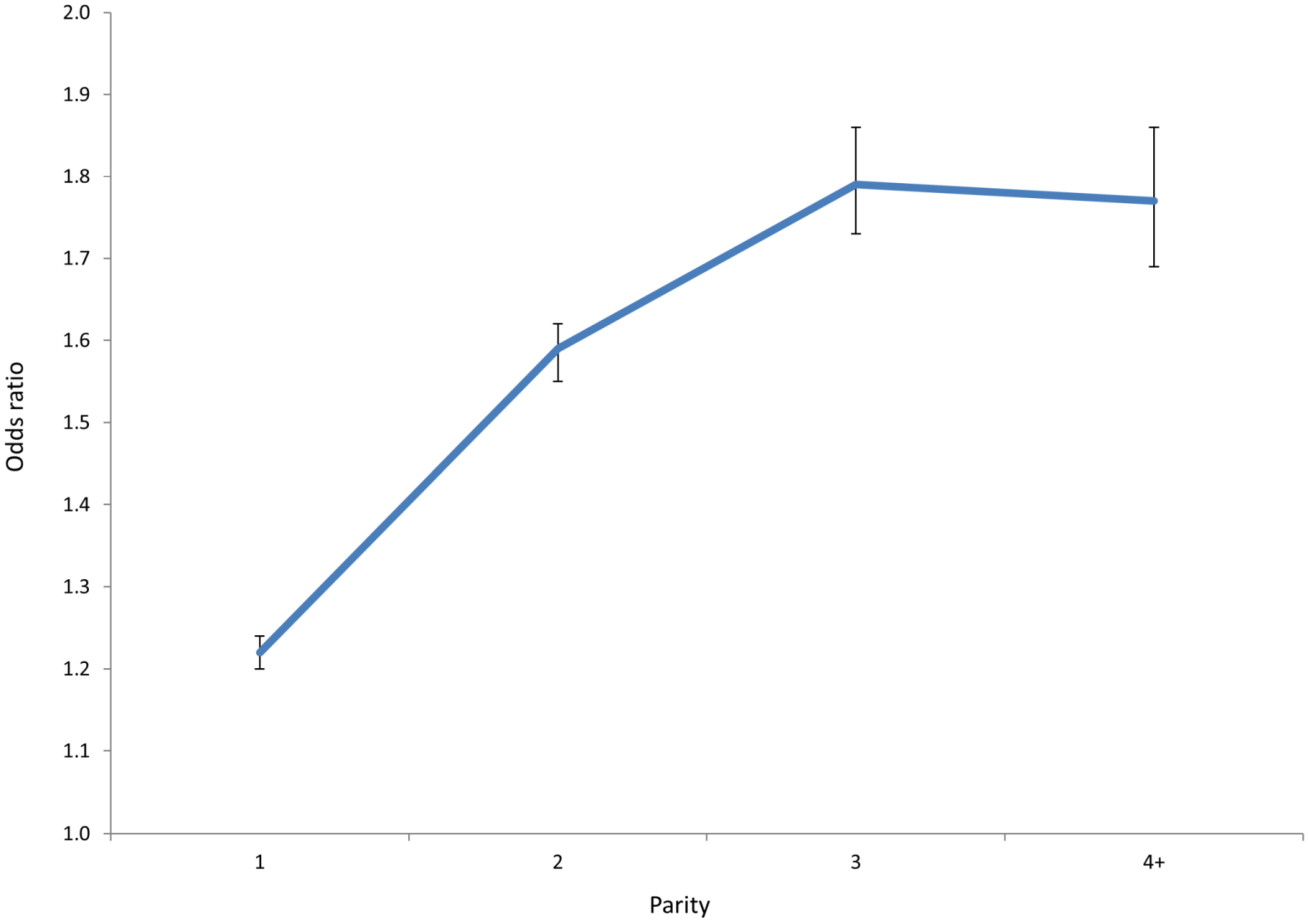
Odds ratios of a long umbilical cord (>90^th^ percentile) on parity, vertical bars represent 95% confidence interval.

**Fig 2:**
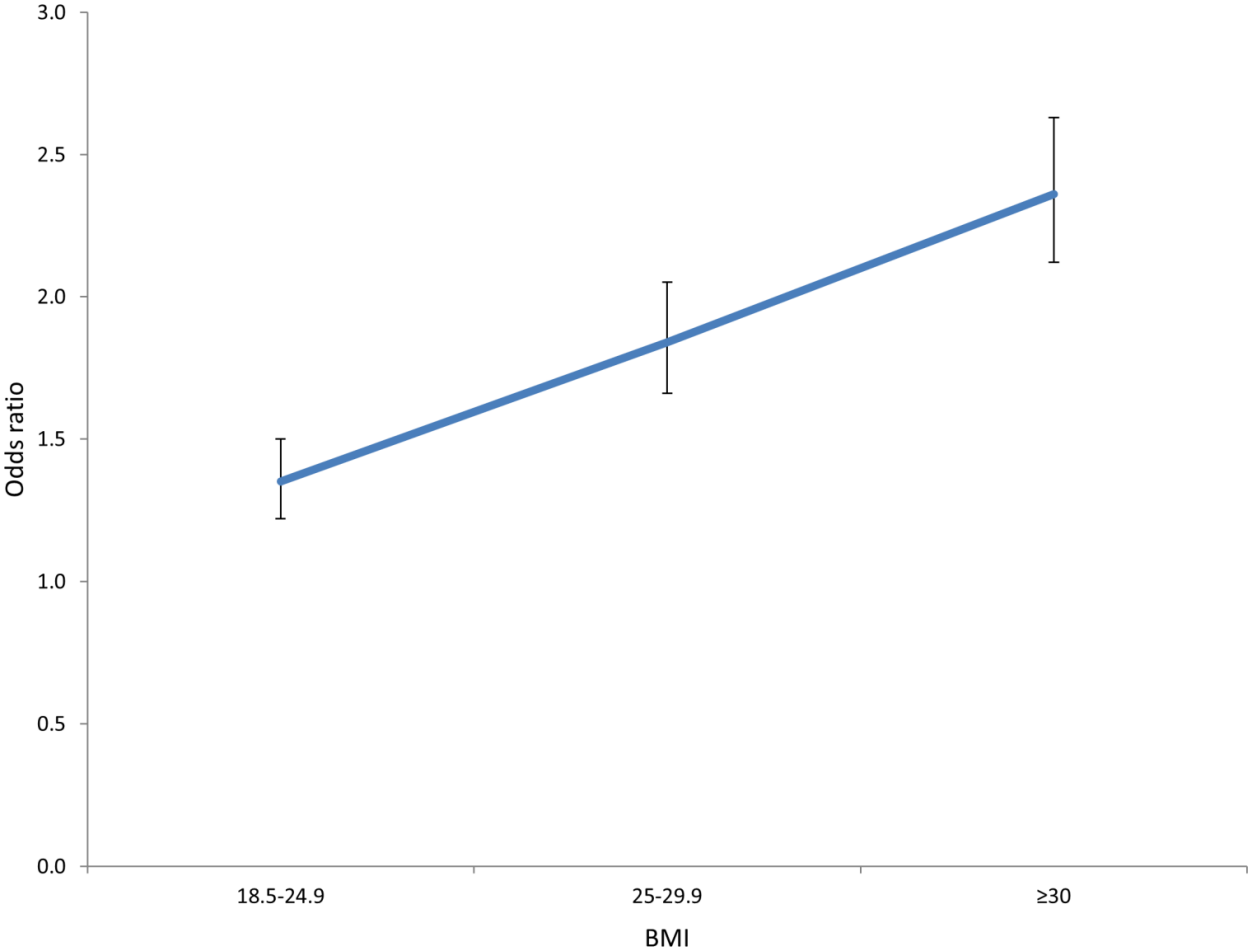
Odds ratios of a long umbilical cord (>90^th^ percentile) on maternal body mass index, vertical bars represent 95% confidence interval.

Maternal parity and BMI significantly increased the risk of developing a long cord in a dose-response pattern (Table 2, Figs 1 and 2).

**Fig 3:**
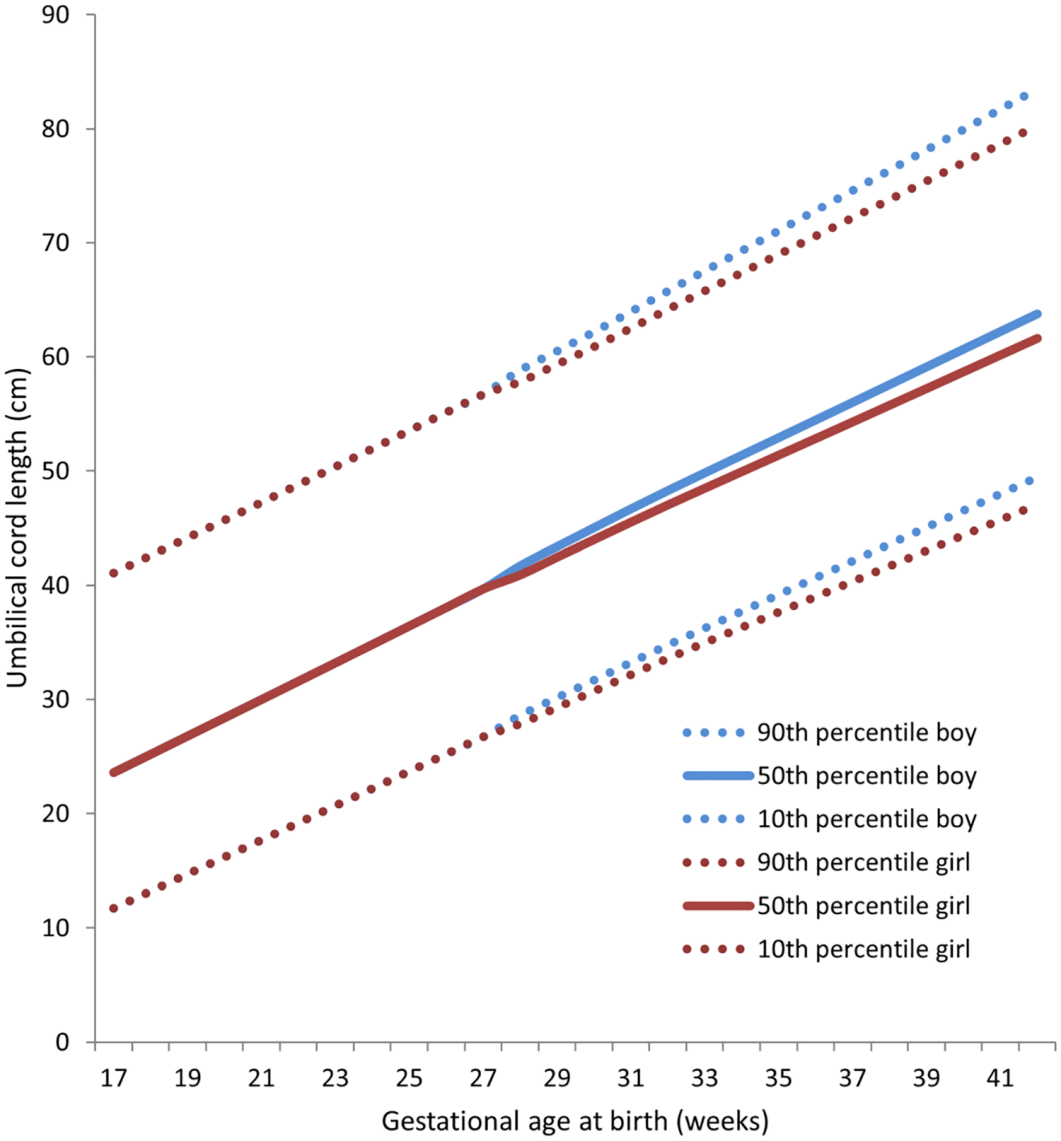
Sex specific percentiles for umbilical cord length based on singleton births in Norway 1999–2013.

Adjusting for maternal age did not change the effects of parity on the risk of a long cord, which implies that parity and not maternal age influence the risk of a long cord. Paternal age had no effect on the risk of an extreme cord length (data not shown). Girls had a lower risk of developing a long cord, cord knots and entanglements than boys (Table 2). Sex differences in cord length were significant after gestational week 28 (Fig 3, and S1 Table).

The effect of daily maternal smoking at the beginning of pregnancy was weak, but showed a tendency to significantly reduce the risk of having a short cord and increase the risk of cord knot and entanglement (Table 2). The risk of a long cord correlated positively to maternal height and BMI at the beginning of pregnancy (Fig 2 and Table 2). Maternal diabetes, particularly pre gestational diabetes mellitus, increased the risk of a long cord (Table 2). For pre gestational diabetes this effect was not significantly altered by including maternal age, parity and BMI in the model, while for gestational diabetes the effect was weaker when BMI was included (adjusted OR (aOR) 1.29, 95%CI 1.17–1.42). Chronic hypertension before pregnancy increased the risk of a long cord and reduced the risk of a short cord (Tables 1 and 2). These findings persisted after including maternal age and parity in the model, but the effects were no longer significant when maternal BMI was included in the model (aOR 1.10, 95%CI 0.91–1.33). Other maternal chronic conditions like asthma, rheumatoid arthritis and epilepsy did not influence the risk of extreme cord length (data not shown). When analyzing the term and preterm group separately, the associations with long or short cord, entanglements, and polyhydramnios did not differ significantly (data not shown).

Conception by ART increased the risk of having a short, but not a long cord. However, the effect almost was abolished when we adjusted for maternal age and parity (aOR 1.09, 95%CI 1.04–1.15).

Placental weight was significantly associated with cord length (Table 2). There was no effect of including maternal age and parity, BMI or diabetes in the model. The relationship to birth weight was similar; birth weight >90^th^ percentile was associated with a doubled risk of having a long cord and reduced risk of having a short cord (Table 2). This was not changed by including maternal diabetes in the model, however, including maternal BMI slightly attenuated the association (aOR 1.66 95%CI 1.58–1.74). Birthweight <10^th^ percentile was associated with reduced risk of a long cord and a doubled risk of a short cord. We tested for co-linearity between placental weight and cord length. Condition Index was low, suggesting that co-linearity is not a concern.

We found a weak positive effect of both poly- and oligohydramnios on the risk of a long cord. This effect persisted after adjustment for maternal age and parity, but when analyzed for the term and preterm group separately, the effect was no longer significant in the preterm group.

#### Risks associated with short or long cord

Tables 3 and 4 show the risks of adverse outcomes associated with the presence of a short or long cord.

**Table 4:** Odds ratios of adverse pregnancy outcomes in pregnancies with a long umbilical cord (>90th sex and parity specific percentile) in the population of singleton births in Norway 1999–2013.

Exposure	Outcome						
Cord>90th percentile	Condition	Yes(n)	Total (n)	%	OR	95% CI	
No	Malformation	32698	728034	4.49			
yes		2858	69062	4.14	0.89	0.85	0.92
No	Placenta previa	2171	728034	0.30			
Yes		127	69062	0.18	0.58	0.48	0.69
No	Abruptio placentae	2679	726465	0.37			
Yes		165	70599	0.23	0.63	0.54	0.74
No	Post-partum haemorrhage^§^	117376	728034	16.12			
Yes		11450	69062	16.58	1.04	1.02	1.07
No	Manual removal of the placenta	10280	728034	1.41			
Yes		839	69062	1.21	0.84	0.78	0.90
No	Preeclampsia	24881	728034	3.42			
Yes		2622	69062	3.80	1.16	1.12	1.21
No	PPROM^¥^	5838	728034	0.80			
Yes		722	69062	1.05	1.26	1.17	1.36
No	Pre-term birth	65105	754586	8.63			
Yes		3957	42510	9.31	1.09	1.05	1.12
No	Transverse lie	2139	728034	0.29			
Yes		188	69062	0.27	0.83	0.71	0.97
No	Breech position	26856	728034	3.69			
Yes		1525	69062	2.21	0.62	0.59	0.65
No	Emergency caesarean	68379	728034	9.39			
Yes		4737	69062	6.86	0.71	0.69	0.74
No	Caesarean delivery	113013	728034	15.52			
yes		7103	69062	10.28	0.62	0.60	0.64
No	Perinatal death	3225	728034	0.44			
Yes		572	69062	0.83	1.83	1.67	2.00
No	Intrauterine death	2363	728034	0.32			
Yes		501	69062	0.73	2.21	2.01	2.44
No	NICU*	55735	728034	7.66			
Yes		5978	69062	8.66	1.11	1.08	1.14
No	5 minute Apgar score<7	66814	775709	8.61			
Yes		2248	21387	10.51	1.25	1.19	1.30

^¥^ Pre-term pre labour rupture of the membranes

* Transferral to neonatal intensive care unit

^§^ Post-partum haemorrhage >500mL

OR; Odds ratio, CI; Confidence Interval

A short cord was associated with a 40% increased risk of the neonate having a major malformation (Table 3, and in S1 List of malformation diagnoses). Fetuses and newborns with a short cord carried increased risk of intrauterine and perinatal death also after including malformations in the model (Table 3). In stratified analyses based on gestational age (term or preterm) this risk was confined to preterm births (aOR of intrauterine death 1.85, 95%CI 1.60–2.14). Also, in preterm births a short cord was associated with an increased risk of a low 5 minutes Apgar score and transferal to intensive care unit (aOR 1.53, 95%CI 1.39–1.68, and 1.30, 95%CI 1.21–1.40, respectively).

Pregnancies with a short cord exhibited increased risk of non-cephalic presentation, in both term and preterm births. Short cord was also associated with increased risk of emergency and all-cause caesarean delivery (Table 3), (in stratified analyses in both term and preterm births). Pregnancies with a short cord also carried an increased risk of placental complications like placenta previa, placental abruption and the need of manual removal of the placenta after birth (Table 3). The associated risk of placental abruption (Table 3) was observed particularly in term births (OR 1.98 (95%CI 1.72–2.73), but was also significant in preterm births (OR 1.35, (95%CI 1.12–1.62). We observed a reduced risk of PPROM and preterm birth when the cord was short. This was also the case for spontaneous preterm birth (data not shown).

On the other hand, pregnancies with a long cord carried a slightly reduced risk for several of the adverse outcomes including malformations, placental abruption, placenta previa, non-cephalic presentation and emergency caesarean delivery (Table 4). A slight increase in the risk of preeclampsia, PPROM, intrauterine and perinatal death, low 5 minute Apgar score, and transferal to NICU was observed in pregnancies with a long cord (Table 4).

#### What influences the risk of entanglement?

The occurrence of umbilical cord entanglement at birth was 20.7% (Table 1). The occurrence declined during the study period (Table 2) and increased with gestational age (Tables 1 and 2). In analyses stratified for gestational age weeks (22–27, 28–36, 37–41, 42+) the risk of entanglement significantly increased if the umbilical cord length was >90th percentile, and correspondingly reduced for cord length <10th percentile in all gestational age groups (OR 2.9–3.1, and OR 0.65–0.22, respectively). In gestational age groups >27 weeks, when cord length was found to differ between the sexes, female fetuses exhibited a significantly lower risk than male fetuses of entanglement (OR 0.86–0.88, 95% CI 0.82–0.91). Including a long or short cord and maternal age and parity in the model did not change this.

Birth weight was significantly associated with entanglements. In SGA (<10th percentile) we found a 22% increased risk for entanglement, and a reduced risk was observed for LGA (>90^th^ percentile). This association was also observed for all groups >27 weeks when we stratified the population according to gestational age. Co-linearity of birth weight and cord length was tested and was not present. After adjustments ART pregnancies carried a slightly lower risk of entanglement. We further explored whether anomalous cord insertion influenced the risk of entanglement. Velamentous and marginal cord insertions were significantly associated with increased risk of entanglement (Table 2). This was present in both preterm and term births (data not shown). Including, maternal age and parity, cord length and neonatal sex to the model did not change these.

While polyhydramnios was not associated with entanglement, oligohydramnios was associated with increased risk of entanglement (OR 1.25, 95%CI 1.21–1.29). Analyzing term and pre term births separately, this association remained significant in the term birth group (OR 1.28, 95%CI 1.24–1.32), whereas the risk of entanglement was reduced in the preterm birth group with oligohydramnios (OR 0.82, 95%CI 0.64–0.96).

#### Risks associated with cord entanglement

In the analyses of associated risks in pregnancies with entanglement at birth results of adjustments for maternal age and parity or other possible confounders are specified in the text when they significantly influenced the association. Births with entanglement carried an increased risk of low 5 minutes Apgar score, intrauterine and perinatal death. However, these risks were confined to term births in analyses stratified based on gestational age (Table 5).

**Table 5:** Odds ratios of adverse pregnancy outcomes in pregnancies with cord entanglement in the population of singleton births in Norway 1999–2013.

Exposure	Outcome						
Entanglement	Condition	Yes (n)	Total (n)	%	OR	95%CI	
No	Malformation	31307	679343	4.61			
Yes		6740	176957	3.81	0.82	0.80	0.84
No	Placenta previa	2208	679343	0.33			
Yes		266	176957	0.15	0.46	0.41	0.52
No	Abruptio placentae	2803	679343	0.41			
Yes		358	176957	0.20	0.49	0.44	0.55
No	Post-partum haemorrhage^§^	112507	679343	16.56			
Yes		25165	176957	14.22	0.84	0.82	0.85
No	Manual removal of the placenta	10303	679343	1.52			
Yes		2306	176957	1.30	0.86	0.82	0.90
No	Preeclampsia	23870	679343	3.51			
Yes		5746	176957	3.25	0.92	0.90	0.95
No	PPROM^¥^	5937	679343	0.87			
Yes		1227	176957	0.69	0.79	0.74	0.84
No	Pre-term birth	40084	679343	5.90			
Yes		6695	176957	3.78	0.63	0.61	0.64
No	Transverse lie	2320	679343	0.34			
Yes		228	176957	0.13	0.38	0.33	0.43
No	Breech position	28135	679343	4.14			
Yes		2577	176957	1.46	0.34	0.33	0.36
No	Emergency caesarean	68937	679343	10.15			
Yes		9880	176957	5.58	0.52	0.51	0.54
No	Caesarean delivery	114749	679343	16.89			
Yes		14127	176957	7.98	0.43	0.42	0.44
No	Perinatal death term	1161	639259	0.18			
Yes		530	170262	0.31	1.72	1.55	1.90
No	Perinatal death pre-term	2414	40084	6.02			
Yes		370	6695	5.53	NS		
No	Intrauterine death term	862	639259	0.13			
Yes		437	170262	0.26	1.94	1.73	2.18
No	Intrauterine death preterm	1778	40084	4.44			
Yes		326	6695	4.87	NS		
No	NICU* term	33252	639259	5.20			
Yes		9258	170262	5.44	1.04	1.02	1.07
No	NICU* pre-term	21603	40084	53.89			
Yes		3135	6695	46.83	0.75	0.72	0.79
No	5 minute Apgar score<7 term	12484	639259	1.95			
Yes		4621	170262	2.71	1.40	1.35	1.45
No	5 minute Apgar score<7 pre-term	5606	40084	13.99			
Yes		773	6695	11.55	0.80	0.74	0.87

^¥^ Preterm pre labor rupture of the membranes

* Transferral to neonatal intensive care unit

^§^ Post partum haemorrhage >500mL

OR; Odds ratio, CI; Confidence Interval

The associated risks for placental complications, preterm and caesarean birth and non-cephalic position, were reduced in births with cord entanglement (Table 5).

#### What influences the risk of cord knots?

The occurrence of cord knot in the total population was 1.32% (Table 1), and the trend was declining during the study period (Table 2). The occurrence did not vary significantly with gestational age at birth (Table 2). Cord knot occurred more often in male than in female fetuses (Tables 1 and 2). Parity significantly increased the risk of cord knot (Table 2).

The strongest risk factor for cord knot was a long umbilical cord (OR 8.42, 95%CI 8.10–8.76). Adding parity and fetal sex to the model did not significantly change this. In pregnancies with a short cord the risk of a knot was markedly reduced (aOR 0.11, 95%CI 0.09–0.13). The risk of a cord knot was increased in polyhydramnios, and adding a long cord to the model significantly reduced the effect of polyhydramnios. Further including maternal age, parity, and neonatal sex, the effect of polyhydramnios on the risk of cord knot disappeared. Also pregnancies with maternal diabetes, and preexisting hypertension (Table 2) were associated with increased risk of cord knot. But also the effect of pre-gestational diabetes on the risk of cord knot disappeared when a long cord was included in the model.

Low placental weight was associated with reduced risk of knot (Table 2), whereas a large placenta was not associated with increased risk of a knot when we included a long cord to the model. In pregnancies after ART we found no difference in the risk of cord knot compared with the rest of the population.

#### Risks associated with cord knot

In the analyses of associated risks in pregnancies with a cord knot at birth adjustments for maternal age and parity did not significantly influence the associations. In the total population cord knot increased the risk of perinatal death (OR 2.65, 95%CI 2.25–3.11). When we stratified the population for gestational age at birth, OR of perinatal death was more than quadrupled at term, and increased by 65% in the preterm group (Table 6).

**Table 6:** Odds ratios of adverse pregnancy outcomes in pregnancies with cord knot in the population of singleton births in Norway 1999–2013.

Exposure	Outcome						
Cord knot	Condition	Yes(n)	Total (n)	%	OR	95%CI	
No	Malformation	37532	845029	4.44			
Yes		515	11271	4.57	NS		
No	Placenta previa	2442	845029	0.29			
Yes		32	11271	0.28	NS		
No	Abruptio placentae	3121	845029	0.37			
Yes		40	11271	0.35	NS		
No	Post-partum haemorrhage^§^	136027	845029	16.10			
Yes		1645	11271	14.59	0.89	0.85	0.94
No	Manual removal of the placenta	12435	845029	1.47			
Yes		174	11271	1.54	NS		
No	Preeclampsia	29164	845029	3.45			
Yes		452	11271	4.01	1.17	1.06	1.29
No	PPROM^¥^	7054	845029	0.83			
Yes		110	11271	0.98	NS		
No	Pre-term birth	46086	845029	5.45			
Yes		693	11271	6.15	1.14	1.05	1.23
No	Transverse lie	2507	845029	0.30			
Yes		41	11271	0.36	NS		
No	Breech position	30435	845029	3.60			
Yes		277	11271	2.46	0.67	0.60	0.76
No	Emergency caesarean	77741	845029	9.20			
Yes		1076	11271	9.55	NS		
No	Caesarean delivery	127127	845029	15.04			
Yes		1749	11271	15.52	NS		
No	Perinatal death term	1600	798943	0.20			
Yes		91	10578	0.86	4.32	3.50	5.35
No	Perinatal death pre-term	2719	46086	5.90			
Yes		65	693	9.38	1.65	1.28	2.14
No	Intrauterine death term	1216	798943	0.15			
Yes		83	10578	0.78	5.19	4.15	6.49
No	Intrauterine death pre-term	2046	46086	4.44			
Yes		58	693	8.37	1.97	1.50	2.58
No	NICU*term birth	41812	798943	5.23			
Yes		698	10578	6.60	1.28	1.18	1.38
No	NICU*pre-term birth	24369	46086	52.88			
Yes		369	693	53.25	NS		
No	5 minute Apgar score<7 term birth	16797	798943	2.10			
Yes		308	10578	2.91	1.40	1.25	1.57
No	5 minute Apgar score<7 pre-term birth	6261	46086	13.59			
Yes		118	693	17.03	1.31	1.07	1.59

^¥^ Pre-term pre-labour rupture of the membranes

* Transferral to neonatal intensive care unit

^§^ Post-partum haemorrhage >500mL

OR; Odds ratio, CI; Confidence Interval

NS; non-significant

Also the risk of intrauterine death and low 5 minutes Apgar score was increased in both term and preterm births with a cord knot (Table 6), whereas the risk of transferal to NICU was increased in the term birth group (Table 6).

#### Combined knot and entanglement

The combination of cord entanglement and knot occurred in 3698 births (0.43%). The occurrence of the combination did not vary significantly between gestational age groups (data not shown), and decreased during the study period, (0.49% in 1999–2003, and 0.37% in 2009–2013), OR 0.75 (95%CI 0.70–0.82). For the total population the risk of perinatal death with combined cord entanglement and knot was increased OR 5.1 (95%CI 4.16–6.27). For gestational age 37–41 and 28–36 weeks the effect of the combination of cord entanglement and knot was more than additive with aORs of perinatal death 9.77 (95%CI 7.57–12.60) and 5.90 (95%CI 3.92–8.87), respectively. Interaction terms between knot and entanglement in the models were significant. Adding birthweight below the 10^th^ percentile to the model did not significantly change these results. There was no significant associated increased risk of perinatal death in births <28 and >41 weeks in births with combined cord knot and entanglement.

#### Recurrence

Finally, we studied the risk of recurrence of extreme cord length, knot and entanglement in a subsequent pregnancy. In our population 289 684 women had at least two births in the register. If the cord was long or short in the first pregnancy, the recurrence risk of long cord or short cord in the subsequent was more than doubled (OR 2.53, (95%CI 2.42–2.64) and OR 2.39, (95%CI 2.29–2.49), respectively). Likewise, cord knot recurred with an OR of 2.64 (95%CI 2.29–3.06). These risks were not influenced by neonatal sex in the second pregnancy, whereas including long cord in the second pregnancy reduced the recurrence risk of a cord knot to OR 1.93 (95%CI 1.52–2.44). Entanglement did also recur with an OR of 4.61 (95%CI 4.50–4.72). Including a long cord in the second birth significantly reduced this to aOR 1.20 (95%CI 1.17–1.23).

## Discussion

The findings of this population based study demonstrate that sex differences in cord length are evident after 27 weeks, that boys have longer cords than girls, and a higher risk of cord knots and entanglement. We also identified risk factors for long and short cord and found parity was a strong factor (Fig 1 and in S1 Table). Placental and birth weight were associated with cord length. Both short and long cords were associated with increased risk of adverse outcome for the fetus and the mother, also after adjustment for important confounders. We found that the combined effect of entanglement and knot on the risk of intrauterine or perinatal death is more than additive, and demonstrate that extreme cord length, (and to a lesser degree cord knot or entanglement) in one pregnancy tend to recur in a subsequent pregnancy of the same woman. We found that in our population the trend of cord knots, entanglements, and long cords during the study period is declining.

Our reference ranges for cord length based on a nationwide registry study do not differ significantly from those of a large Finnish hospital based study [10]. The cord length at birth increases linearly through gestation, and continues to increase also beyond 40 weeks (Fig 3). Georgiadis *et al*. [10] found a possible association of a short cord to abruption of the placenta, and our study is large enough to confirm that a short cord is associated with a 50% increased risk of abruption in the total population (Table 3), and a doubled risk at term. Polyhydramnios was in our study associated with an increased risk of cord knot, which corroborates earlier findings [4]. We find a slightly higher occurrence of cord knot (1.3% vs 1.2%), but lower associated risks in pregnancies with a cord knot than those reported in a hospital study [11]. Both risk factors for short cord, cord knot and associated risks of adverse outcomes including maternal complications and stillbirth identified in our study compares well with other studies [9, 11, 12]. In addition, our study finds that the risk of stillbirth in pregnancies with cord knot and entanglement is higher in term than preterm births (Tables 5 and 6). In contrast to a case-control study [9], we find increased risk of non-cephalic position when the cord is short, and reduced risk when the cord is long (Tables 3 and 4). Further, we find an increased risk of caesarean delivery in short cord pregnancies, which is in opposition to the case-control study [9]. The observed differences may be due to differences in the definition of a short cord, different populations and study design.[1–3] Strength of the study is that it is population based, which reduces selection bias, and its large size, which makes it possible to study exposures and outcomes with a low incidence. The data from the MBRN also makes it possible to calculate recurrence risk and trend. Several of the variables in the MBRN have been validated [13–15], and our recent study of midwives measurements, classification and notifications to the MBRN on placenta and cord findings suggest these data are valid [16]. We consider it a strength that the present study is comprehensive by assessing risk factors and associated risks for both a short and a long cord, knots and entanglements in the same study.

Amniotic fluid amount was only estimated clinically at birth and was not verified by ultrasound. Therefore our results regarding the association between poly- and oligohydramnios with cord length, knot and entanglement should be interpreted with caution. The only paternal characteristic that was available in our study was age, and the effect of paternal age was abolished when adjusted for maternal age or parity. We also did not have access to information of socioeconomic or ethnic factors that may influence cord length or other outcomes or exposures.

Because of lacking information on number of loops and of which body parts the cord was entangled, we were unable to study whether nuchal entanglement or the number of loops influence risks. Our study does not contain information of whether (nuchal) entanglements were identified prenatally. A fetus may entangle or untangle during the rotational movements during delivery. Thus, this needs to be studied in a clinical setting comparing ultrasound identification or exclusion of cord entanglement directly before delivery. A previous study of high-risk pregnancies found that entanglement was less common in caesarean than vaginal births, indicating that entanglement may occur during delivery [17].

Information about umbilical cord coiling or other features of umbilical cord are not available in the register. Because of practical circumstances clamping and cutting of the cord may be performed in to stages in cesarean delivery, (clamping by the surgeon and thereafter cutting by the midwife). Thus, a small part of the cord may be lost to measure, and we cannot entirely rule out a systematic bias towards measuring a shorter cord when the delivery was by caesarean. However, when analyzing caesarean and vaginal births separately the risks estimates did not differ significantly. We cannot infer from our study whether cord entanglement contributes to growth restriction, or the other way around. The same applies for the association of oligohydramnios and entanglement.

The identified risk factors had opposite effect on the risk of a long compared to the risk of a short cord, which supports the biological plausibility of our findings (Tables 1 and 2). Several examples of dose-response relations in our study further lend support to this, for example the risk of a long cord increased significantly with BMI class (Fig 1, panel B). The fact that a short cord carried increased risk of placental complications and a long cord was associated with a reduced risk for these complications (Tables 3 and 4) suggest that the development of a short cord and abnormal placentation is linked.

We provide population based empirical reference ranges (fetal sex and parity specific) for umbilical cord length (Fig 3 and in S1 Table). However, it is important to bear in mind that these reference ranges are based on cord length in born individuals and they may not be representative for those still in utero.

Cord compression in knots or entanglements may reduce umbilical blood flow [18]. An experimental study of cord compression in fetal sheep (0.6 gestation) shows that compression of the umbilical cord alters the distribution of the umbilical and systemic blood flow [19, 20] and the fetal responses to this challenge differ with gestational age [21]. In late pregnancy less of the fetal cardiac output is directed to the placenta [22], but the fetal demand increases, which in turn increase the vulnerability for cord accident and obstruction of umbilical flow in late pregnancy. This is in line with our finding that the risk of intrauterine death is quadrupled at term when an umbilical cord knot is present and 10 times increased when the cord is both entangled and has a knot. However, the finding that the combination of cord knot and entanglement was associated with increased risk of stillbirth only in the term birth group may be due to low numbers in the pre- and post-term groups.

One may argue that it is unclear what the clinician should do with the information of the umbilical cord length, entanglements and knots. Prenatal identification of these abnormalities with ultrasound is hampered with low sensitivity and specificity, and the results may cause unnecessary worries and frustration for the mother and the clinician [23, 24]. However, prenatal identification of conditions that are associated with compressed umbilical cord are suggested in the literature to be offered close follow-up [25]. The increased risk of recurrence of cord anomalies found in this study may contribute to justify extra clinical follow-up in a pregnancy following one with anomalous cord or cord accidents.

Since cord anomalies are important risk factors for stillbirth the results of the present study support that the umbilical cord and placenta should be given special attention by perinatal pathologists in these tragic events [26].

Large-scale public health data utilized in epidemiological studies are important steps in the way to increase the insight to the development in human (fetal) biology. Genetic and environmental factors influence the development of the cord. A twin study suggests that cord length is influenced by heritable factors, whereas their interpretation was that twisting and cord insertion is strongly influenced by nongenetic factors [27]. The results from a previous study (focusing on cord insertion site) and the present found that fetal sex has a strong influence, and increased risk of recurrence of cord length and insertion suggest that genetic and persisting environmental factors influence the development of the cord and placenta in singletons [4]. Maternal diabetes significantly influence the expression of genes in the umbilical cord and alters the umbilical vessel phenotype, with possible long term consequences for the neonate [28].

The fact that cord knots and entanglements occurred more often in male fetuses may be attributable to longer cords in boys than girls. Although disputed [9], the “stretch hypothesis” which says that tensile force is an important determinant for cord length [29] suggests that an active fetus develop a longer cord. There have also been raised theories of male fetuses exhibiting a higher level of activity in the womb [30, 31]. This fits with our observation that boys had longer cords and increased risk of entanglements and knots, also after adjusting for long cord and parity. The differential effect of sex on cord length is supported by the finding that boys are more active in the womb, and show larger response to vibroacoustic stimulation than girls from 31 weeks [32–34]. Our findings of differences between girls and boys are consistent with previous findings that the placenta has sex differential features from early gestation: Sex-chromosome genes are differently expressed in human male and female placentas, and male placentas are more responsive to changes in maternal environment than female [35, 36]. However, an ultrasound study of umbilical constriction at the abdominal wall inlet found that the degree of constriction was positively associated with a longer cord only in female fetuses [37].

It has been shown that fetuses in breech position had reduced body movements in response to vibroacoustic stimulation compared with fetuses in cephalic position [38]. This is in line with our observation that breech position was associated with reduced occurrence of a long cord, cord knot and entanglement, and increased risk of a short cord, which further supports the theory that fetal activity influence cord length, knots and entanglement.

## Conclusions

Our population study indicates that cord length was determined by both fetal and maternal factors in singletons, and that fetal sex and parity were important determinants. There was an increased risk of recurrence of extreme cord length, knots and entanglement. Extreme cord length, entanglement and particularly cord knot were associated with increased risk of adverse outcomes including a more than doubled risk of perinatal death. The combination of cord knot and entanglement seem to exhibit more than additive effect on the risk of perinatal death, an almost 10 times increased risk at term. We provide population based parity and sex specific reference ranges for umbilical cord length.

## Supporting information

S1 Table Gestational age (weeks), parity (0–1+) and sex specific empirical umbilical cord length (cm) percentiles. (DOCX)

S1 List Malformation diagnoses. (DOCX)

## Abbreviations

aOR: adjusted Odds Ratio
ART: Assisted reproductive technology
CI: Confidence interval
MBRN: Medical birth registry of Norway
OR: Odds Ratio

## References

[1] Baergen RN, Malicki D, Behling C, Benirschke K. Morbidity, mortality, and placental pathology in excessively long umbilical cords: retrospective study. PediatrDevPathol. 2001;4(2):144–53. doi: 10.1007/s100240010135 11178630 10.1007/s100240010135

[2] Vintzileos AM, Ananth CV, Smulian JC. Using ultrasound in the clinical management of placental implantation abnormalities. American Journal of Obstetrics and Gynecology The Human Placenta; 10/20152015. p. S70–S7. 10.1016/j.ajog.2015.05.059 26428505

[3] Ebbing C, Johnsen SL, Albrechtsen S, Sunde ID, Vekseth C, Rasmussen S. Velamentous or marginal cord insertion and the risk of spontaneous preterm birth, prelabor rupture of the membranes, and anomalous cord length, a population-based study. Acta Obstet Gynecol Scand. 2016 doi: 10.1111/aogs.13035 . 27696344 10.1111/aogs.13035

[4] Ebbing C, Kiserud T, Johnsen SL, Albrechtsen S, Rasmussen S. Prevalence, risk factors and outcomes of velamentous and marginal cord insertions: a population-based study of 634,741 pregnancies. PLoSOne. 2013;8(7):e70380 doi: 10.1371/journal.pone.0070380 23936197 10.1371/journal.pone.0070380 PMC3728211

[5] Weiner E, Fainstein N, Schreiber L, Sagiv R, Bar J, Kovo M. The association between umbilical cord abnormalities and the development of non-reassuring fetal heart rate leading to emergent cesarean deliveries. J Perinatol. 2015;35(11):919–23. doi: 10.1038/jp.2015.102 . 26291780 10.1038/jp.2015.102

[6] Taweevisit M, Thorner PS. Massive fetal thrombotic vasculopathy associated with excessively long umbilical cord and fetal demise: case report and literature review. PediatrDevPathol. 2010;13(2):112–5. doi: 10.2350/09-07-0680-CR.1 19888870 10.2350/09-07-0680-CR.1

[7] Olaya C, Bernal JE. Clinical associations to abnormal umbilical cord length in Latin American newborns. JNeonatal PerinatalMed. 2015;8(3):251–6. doi: 10.3233/NPM-15915056 26485559 10.3233/NPM-15915056

[8] Georgiadis L, Keski-Nisula L, Harju M, Raisanen S, Georgiadis S, Hannila ML, et al Umbilical cord length in singleton gestations: A Finnish population-based retrospective register study. Placenta. 2014 doi: 10.1016/j.placenta.2014.02.001 24560495 10.1016/j.placenta.2014.02.001

[9] Krakowiak P, Smith EN, de BG, Lydon-Rochelle MT. Risk factors and outcomes associated with a short umbilical cord. ObstetGynecol. 2004;103(1):119–27. 10.1097/01.AOG.0000102706.84063.C7 14704255

[10] Georgiadis L, Keski-Nisula L, Harju M, Raisanen S, Georgiadis S, Hannila ML, et al Umbilical cord length in singleton gestations: a Finnish population-based retrospective register study. Placenta. 2014;35(4):275–80. doi: 10.1016/j.placenta.2014.02.001 24560495 10.1016/j.placenta.2014.02.001

[11] Raisanen S, Georgiadis L, Harju M, Keski-Nisula L, Heinonen S. True umbilical cord knot and obstetric outcome. IntJGynaecolObstet. 2013;122(1):18–21. doi: 10.1016/j.ijgo.2013.02.012 23523334 10.1016/j.ijgo.2013.02.012

[12] Airas U, Heinonen S. Clinical significance of true umbilical knots: a population-based analysis. Am J Perinatol. 2002;19(3):127–32. doi: 10.1055/s-2002-25311 . 12012287 10.1055/s-2002-25311

[13] Baghestan E, Bordahl PE, Rasmussen SA, Sande AK, Lyslo I, Solvang I. A validation of the diagnosis of obstetric sphincter tears in two Norwegian databases, the Medical Birth Registry and the Patient Administration System. Acta ObstetGynecolScand. 2007;86(2):205–9. doi: 10.1080/00016340601111364 17364284 10.1080/00016340601111364

[14] Engeland A, Bjorge T, Daltveit AK, Vollset SE, Furu K. Validation of disease registration in pregnant women in the Medical Birth Registry of Norway. Acta ObstetGynecolScand. 2009;88(10):1083–9. doi: 10.1080/00016340903128454 19657758 10.1080/00016340903128454

[15] Rasmussen S, Albrechtsen S, Irgens LM, Dalaker K, Maartmann-Moe H, Vlatkovic L, et al Unexplained antepartum fetal death in Norway, 1985–97: diagnostic validation and some epidemiologic aspects. Acta ObstetGynecolScand. 2003;82(2):109–15. 10.1034/j.1600-0412.2003.00009.x 12648170

[16] Sunde ID, Vekseth C, Rasmussen S, Mahjoob E, Collett K, Ebbing C. Placenta, cord and membranes: a dual center validation study of midwives’ classifications and notifications to the Medical Birth Registry of Norway. Acta Obstetricia et Gynecologica Scandinavica. n/a–n/a. doi: 10.1111/aogs.13164 28481411 10.1111/aogs.13164

[17] Vintzileos AM, Egan JFX, Rodis JF, Campbell WA, Wolf EJ, Balducci J. Obstetrical Factors Associated with Nuchal Cord in a High-Risk Population. Journal of Maternal-Fetal Medicine. 1992;1(4):196–201. doi: 10.3109/14767059209161918

[18] Gembruch U, Baschat AA. True knot of the umbilical cord: transient constrictive effect to umbilical venous blood flow demonstrated by Doppler sonography. Ultrasound Obstet Gynecol. 1996;8(1):53–6. Epub 1996/07/01. doi: 10.1046/j.1469-0705.1996.08010053.x . 8843621 10.1046/j.1469-0705.1996.08010053.x

[19] Itskovitz J, LaGamma EF, Rudolph AM. Effects of cord compression on fetal blood flow distribution and O2 delivery. The American journal of physiology. 1987;252(1 Pt 2):H100–9. Epub 1987/01/01. doi: 10.1152/ajpheart.1987.252.1.H100 . 3101514 10.1152/ajpheart.1987.252.1.H100

[20] Guzikowski W, Kowalczyk D, Wiecek J. Diagnosis of true umbilical cord knot. Archives of medical science: AMS. 2014;10(1):91–5. Epub 2014/04/05. doi: 10.5114/aoms.2013.33068 . 24701220 10.5114/aoms.2013.33068 PMC3953963

[21] Jensen A, Roman C, Rudolph AM. Effects of reducing uterine blood flow on fetal blood flow distribution and oxygen delivery. Journal of developmental physiology. 1991;15(6):309–23. Epub 1991/06/01. . 1753071

[22] Kiserud T, Ebbing C, Kessler J, Rasmussen S. Fetal cardiac output, distribution to the placenta and impact of placental compromise. Ultrasound in Obstetrics & Gynecology. 2006;28(2):126–36. 16826560 10.1002/uog.2832

[23] Sepulveda W, Shennan AH, Bower S, Nicolaidis P, Fisk NM. True knot of the umbilical cord: a difficult prenatal ultrasonographic diagnosis. Ultrasound Obstet Gynecol. 1995;5(2):106–8. Epub 1995/02/01. doi: 10.1046/j.1469-0705.1995.05020106.x . 7719859 10.1046/j.1469-0705.1995.05020106.x

[24] Sepulveda W, Wong AE, Gomez L, Alcalde JL. Improving Sonographic Evaluation of the Umbilical Cord at the Second-Trimester Anatomy Scan. Journal of Ultrasound in Medicine. 2009;28(6):831–5. 19470827 10.7863/jum.2009.28.6.831

[25] Sherer DM, Manning FA. Prenatal ultrasonographic diagnosis of conditions associated with potential umbilical cord compression. Am J Perinatol. 1999;16(9):445–58. Epub 2000/04/25. . 10774759 10.1055/s-1999-6807

[26] Salafia CM, Vintzileos AM. Why all placentas should be examined by a pathologist in 1990. Am J Obstet Gynecol. 1990;163(4 Pt 1):1282–93. Epub 1990/10/01. . 2121035 10.1016/0002-9378(90)90708-f

[27] Antoniou EE, Derom C, Thiery E, Fowler T, Southwood TR, Zeegers MP. The influence of genetic and environmental factors on the etiology of the human umbilical cord: the East Flanders prospective twin survey. BiolReprod. 2011;85(1):137–43. doi: 10.1095/biolreprod.110.088807 21325690 10.1095/biolreprod.110.088807

[28] Koskinen A, Lehtoranta L, Laiho A, Laine J, Kaapa P, Soukka H. Maternal diabetes induces changes in the umbilical cord gene expression. Placenta. 2015;36(7):767–74. Epub 2015/05/04. doi: 10.1016/j.placenta.2015.04.004 . 25935091 10.1016/j.placenta.2015.04.004

[29] Miller ME, Jones MC, Smith DW. Tension: the basis of umbilical cord growth. JPediatr. 1982;101(5):844. 7131174 10.1016/s0022-3476(82)80344-2

[30] Verbruggen SW, Loo JH, Hayat TT, Hajnal JV, Rutherford MA, Phillips AT, et al Modeling the biomechanics of fetal movements. Biomechanics and modeling in mechanobiology. 2016;15(4):995–1004. Epub 2015/11/05. doi: 10.1007/s10237-015-0738-1 26534772 10.1007/s10237-015-0738-1 PMC4945693

[31] Almli CR, Ball RH, Wheeler ME. Human fetal and neonatal movement patterns: Gender differences and fetal-to-neonatal continuity. Dev Psychobiol. 2001;38(4):252–73. Epub 2001/04/25. . 11319731 10.1002/dev.1019

[32] Buss C, Davis EP, Class QA, Gierczak M, Pattillo C, Glynn LM, et al Maturation of the human fetal startle response: evidence for sex-specific maturation of the human fetus. Early Hum Dev. 2009;85(10):633–8. doi: 10.1016/j.earlhumdev.2009.08.001 19726143 10.1016/j.earlhumdev.2009.08.001 PMC2767415

[33] Glynn LM, Sandman CA. Sex moderates associations between prenatal glucocorticoid exposure and human fetal neurological development. Developmental science. 2012;15(5):601–10. Epub 2012/08/29. doi: 10.1111/j.1467-7687.2012.01159.x . 22925508 10.1111/j.1467-7687.2012.01159.x

[34] Hepper PG, Dornan JC, Lynch C. Sex differences in fetal habituation. Developmental science. 2012;15(3):373–83. Epub 2012/04/12. doi: 10.1111/j.1467-7687.2011.01132.x . 22490177 10.1111/j.1467-7687.2011.01132.x

[35] Charil A, Laplante DP, Vaillancourt C, King S. Prenatal stress and brain development. Brain research reviews. 2010;65(1):56–79. doi: 10.1016/j.brainresrev.2010.06.002 . 20550950 10.1016/j.brainresrev.2010.06.002

[36] Gabory A, Roseboom TJ, Moore T, Moore LG, Junien C. Placental contribution to the origins of sexual dimorphism in health and diseases: sex chromosomes and epigenetics. Biology of sex differences. 2013;4(1):5 doi: 10.1186/2042-6410-4-5 23514128 10.1186/2042-6410-4-5 PMC3618244

[37] Skulstad SM, Rasmussen S, Seglem S, Svanaes RH, Aareskjold HM, Kiserud T. The effect of umbilical venous constriction on placental development, cord length and perinatal outcome. Early Hum Dev. 2005;81(4):325–31. doi: 10.1016/j.earlhumdev.2004.07.006 . 15814216 10.1016/j.earlhumdev.2004.07.006

[38] Van der Meulen JA, Davies GA, Kisilevsky BS. Fetal sensory-elicited body movements differ in breech compared to cephalic position. Dev Psychobiol. 2008;50(5):530–4. Epub 2008/06/14. doi: 10.1002/dev.20306 . 18551470 10.1002/dev.20306

