# Supplementary material for: Extreme umbilical cord lengths, cord knot and entanglement: Risk factors and risk of adverse outcomes, a population-based study
Source: PLoS One. 2018 Mar 27;13(3):e0194814. doi: 10.1371/journal.pone.0194814 (PMC5870981; doi:10.1371/journal.pone.0194814)
Supplement: S1 Table — (DOCX) [file pone.0194814.s001.docx]

**S1 Table** Gestational age (weeks), parity (0. 1+) and gender specific empirical umbilical cord length (cm) percentiles

|  | **Para 0 girls** | | |  |  |  | **Para 0 boys** | | | |  |  | **Para 1+girls** | | |  |  |  | **Para 1+ boys** | | |  |  |
| --- | --- | --- | --- | --- | --- | --- | --- | --- | --- | --- | --- | --- | --- | --- | --- | --- | --- | --- | --- | --- | --- | --- | --- |
| **Gestational age (Weeks)** | **Percentiles** | | | | | | | | | | | | | | | | | | | | | | |
|  | **50** | **5** | **10** | **90** | **95** |  | **50** | **5** | **10** | **90** | **95** |  | **50** | **5** | **10** | **90** | **95** |  | **50** | **5** | **10** | **90** | **95** |
| 17 | 23.6 | 9.3 | 11.7 | 41.1 | 48.4 |  | 23.6 | 9.3 | 11.7 | 41.1 | 48.4 |  | 23.6 | 9.3 | 11.7 | 41.1 | 48.4 |  | 23.6 | 9.3 | 11.7 | 41.1 | 48.4 |
| 18 | 25.2 | 10.7 | 13.2 | 42.6 | 49.9 |  | 25.2 | 10.7 | 13.2 | 42.6 | 49.9 |  | 25.2 | 10.7 | 13.2 | 42.6 | 49.9 |  | 25.2 | 10.7 | 13.2 | 42.6 | 49.9 |
| 19 | 26.8 | 12.1 | 14.7 | 44.1 | 51.4 |  | 26.8 | 12.1 | 14.7 | 44.1 | 51.4 |  | 26.8 | 12.1 | 14.7 | 44.1 | 51.4 |  | 26.8 | 12.1 | 14.7 | 44.1 | 51.4 |
| 20 | 28.4 | 13.5 | 16.2 | 45.7 | 52.9 |  | 28.4 | 13.5 | 16.2 | 45.7 | 52.9 |  | 28.4 | 13.5 | 16.2 | 45.7 | 52.9 |  | 28.4 | 13.5 | 16.2 | 45.7 | 52.9 |
| 21 | 30.0 | 14.9 | 17.7 | 47.2 | 54.4 |  | 30.0 | 14.9 | 17.7 | 47.2 | 54.4 |  | 30.0 | 14.9 | 17.7 | 47.2 | 54.4 |  | 30.0 | 14.9 | 17.7 | 47.2 | 54.4 |
| 22 | 31.6 | 16.3 | 19.2 | 48.8 | 55.9 |  | 31.6 | 16.3 | 19.2 | 48.8 | 55.9 |  | 31.6 | 16.3 | 19.2 | 48.8 | 55.9 |  | 31.6 | 16.3 | 19.2 | 48.8 | 55.9 |
| 23 | 33.2 | 17.7 | 20.7 | 50.4 | 57.4 |  | 33.2 | 17.7 | 20.7 | 50.4 | 57.4 |  | 33.2 | 17.7 | 20.7 | 50.4 | 57.4 |  | 33.2 | 17.7 | 20.7 | 50.4 | 57.4 |
| 24 | 34.8 | 19.2 | 22.2 | 51.9 | 58.9 |  | 34.8 | 19.2 | 22.2 | 51.9 | 58.9 |  | 34.8 | 19.2 | 22.2 | 51.9 | 58.9 |  | 34.8 | 19.2 | 22.2 | 51.9 | 58.9 |
| 25 | 36.4 | 20.6 | 23.7 | 53.5 | 60.5 |  | 36.4 | 20.6 | 23.7 | 53.5 | 60.5 |  | 36.4 | 20.6 | 23.7 | 53.5 | 60.5 |  | 36.4 | 20.6 | 23.7 | 53.5 | 60.5 |
| 26 | 38.1 | 22.0 | 25.2 | 55.1 | 62.0 |  | 38.1 | 22.0 | 25.2 | 55.1 | 62.0 |  | 38.1 | 22.0 | 25.2 | 55.1 | 62.0 |  | 38.1 | 22.0 | 25.2 | 55.1 | 62.0 |
| 27 | 39.7 | 23.4 | 26.7 | 56.7 | 63.6 |  | 39.7 | 23.4 | 26.7 | 56.7 | 63.6 |  | 39.7 | 23.4 | 26.7 | 56.7 | 63.6 |  | 39.7 | 23.4 | 26.7 | 56.7 | 63.6 |
| 28 | 40.5 | 24.0 | 27.5 | 57.3 | 63.9 |  | 41.3 | 25.0 | 28.3 | 58.1 | 65.1 |  | 41.3 | 24.7 | 28.2 | 58.3 | 65.2 |  | 42.1 | 25.6 | 28.9 | 59.6 | 65.1 |
| 29 | 42.0 | 25.3 | 28.9 | 58.7 | 65.3 |  | 42.9 | 26.4 | 29.8 | 59.7 | 66.6 |  | 43.0 | 26.1 | 29.8 | 59.9 | 66.8 |  | 43.9 | 27.1 | 30.5 | 61.3 | 66.6 |
| 30 | 43.4 | 26.6 | 30.2 | 60.2 | 66.7 |  | 44.5 | 27.9 | 31.3 | 61.2 | 68.3 |  | 44.5 | 27.6 | 31.2 | 61.6 | 68.4 |  | 45.6 | 28.6 | 32.1 | 63.1 | 68.3 |
| 31 | 44.9 | 28.0 | 31.6 | 61.7 | 68.2 |  | 46.1 | 29.3 | 32.8 | 62.9 | 70.0 |  | 46.1 | 29.0 | 32.8 | 63.3 | 70.1 |  | 47.3 | 30.2 | 33.7 | 65.0 | 70.0 |
| 32 | 46.3 | 29.3 | 32.9 | 63.3 | 69.8 |  | 47.6 | 30.7 | 34.3 | 64.6 | 71.7 |  | 47.7 | 30.5 | 34.3 | 65.0 | 71.8 |  | 49.0 | 31.7 | 35.3 | 66.9 | 71.7 |
| 33 | 47.7 | 30.6 | 34.3 | 64.9 | 71.4 |  | 49.0 | 32.1 | 35.7 | 66.3 | 73.4 |  | 49.2 | 31.9 | 35.7 | 66.6 | 73.5 |  | 50.6 | 33.2 | 36.8 | 68.8 | 73.4 |
| 34 | 49.1 | 31.9 | 35.6 | 66.5 | 73.0 |  | 50.5 | 33.5 | 37.1 | 67.9 | 75.1 |  | 50.8 | 33.2 | 37.1 | 68.3 | 75.1 |  | 52.3 | 34.6 | 38.3 | 70.7 | 75.1 |
| 35 | 50.5 | 33.2 | 36.9 | 68.0 | 74.6 |  | 51.9 | 34.8 | 38.6 | 69.5 | 76.8 |  | 52.3 | 34.6 | 38.4 | 69.9 | 76.8 |  | 53.9 | 36.1 | 39.8 | 72.6 | 76.8 |
| 36 | 51.9 | 34.5 | 38.2 | 69.6 | 76.2 |  | 53.4 | 36.2 | 40.0 | 71.1 | 78.5 |  | 53.8 | 36.0 | 39.8 | 71.6 | 78.5 |  | 55.5 | 37.5 | 41.3 | 74.5 | 78.5 |
| 37 | 53.3 | 35.8 | 39.5 | 71.1 | 77.8 |  | 54.9 | 37.6 | 41.4 | 72.8 | 80.2 |  | 55.3 | 37.3 | 41.2 | 73.3 | 80.1 |  | 57.2 | 38.9 | 42.9 | 76.4 | 80.2 |
| 38 | 54.7 | 37.1 | 40.8 | 72.7 | 79.4 |  | 56.3 | 38.9 | 42.8 | 74.4 | 81.9 |  | 56.8 | 38.7 | 42.6 | 74.9 | 81.8 |  | 58.8 | 40.4 | 44.4 | 78.3 | 81.9 |
| 39 | 56.1 | 38.4 | 42.1 | 74.2 | 81.1 |  | 57.8 | 40.3 | 44.3 | 76.1 | 83.7 |  | 58.3 | 40.1 | 43.9 | 76.6 | 83.5 |  | 60.4 | 41.8 | 45.9 | 80.2 | 83.7 |
| 40 | 57.5 | 39.7 | 43.4 | 75.8 | 82.7 |  | 59.3 | 41.7 | 45.7 | 77.7 | 85.4 |  | 59.9 | 41.4 | 45.3 | 78.3 | 85.2 |  | 62.1 | 43.2 | 47.4 | 82.1 | 85.4 |
| 41 | 58.9 | 41.0 | 44.7 | 77.4 | 84.4 |  | 60.7 | 43.1 | 47.1 | 79.4 | 87.1 |  | 61.4 | 42.8 | 46.7 | 79.9 | 86.9 |  | 63.7 | 44.7 | 48.9 | 84.1 | 87.1 |
| 42 | 60.3 | 42.3 | 46.1 | 79.0 | 86.0 |  | 62.2 | 44.4 | 48.5 | 81.0 | 88.9 |  | 62.9 | 44.2 | 48.1 | 81.6 | 88.6 |  | 65.3 | 46.1 | 50.4 | 86.0 | 88.9 |
