# Supplementary material for: Extreme umbilical cord lengths, cord knot and entanglement: Risk factors and risk of adverse outcomes, a population-based study
Source: PLoS One. 2018 Mar 27;13(3):e0194814. doi: 10.1371/journal.pone.0194814 (PMC5870981; doi:10.1371/journal.pone.0194814)
Supplement: S1 List — (DOCX) [file pone.0194814.s002.docx]

**S2: List of malformations**

ICD10 diagnoses: Q000-Q139; Q048; Q054; Q059; Q078-Q079; Q111-Q112; Q120; Q130-Q131; Q15; Q172; Q180 Q182; Q188; Q200-Q289; Q30; Q318; Q330; Q333; Q350-Q379; Q39; Q400; Q408; Q410-Q459; Q500-Q529; Q54; Q557-Q558; Q56; Q601; Q606; Q62; Q634; Q640-Q641; Q643; Q648; Q660; Q680; Q71; Q743; Q75; Q77; Q780; Q790; Q792-Q793; Q813; Q850-Q851; Q89; Q9; P835
